# Inhibiting WEE1 Augments the Antitumor Efficacy of Cisplatin in Urothelial Carcinoma by Enhancing the DNA Damage Process

**DOI:** 10.3390/cells12111471

**Published:** 2023-05-25

**Authors:** Yu-Li Su, Ling-Yi Xiao, Shih-Yu Huang, Chia-Che Wu, Li-Chung Chang, Yi-Hua Chen, Hao-Lun Luo, Chun-Chieh Huang, Ting-Ting Liu, Jei-Ming Peng

**Affiliations:** 1 Division of Hematology Oncology, Department of Internal Medicine, Kaohsiung Chang Gung Memorial Hospital and Chang Gung University, College of Medicine, Kaohsiung 83301, Taiwan; 2 Genomic & Proteomic Core Laboratory, Department of Medical Research, Kaohsiung Chang Gung Memorial Hospital, Kaohsiung 83301, Taiwan; 3 Department of Urology, Kaohsiung Chang Gung Memorial Hospital and Chang Gung University, College of Medicine, Kaohsiung 83301, Taiwan; 4 Department of Radiation Oncology, Kaohsiung Chang Gung Memorial Hospital and Chang Gung University, College of Medicine, Kaohsiung 83301, Taiwan; 5 Department of Pathology, Kaohsiung Chang Gung Memorial Hospital and Chang Gung University, College of Medicine, Kaohsiung 83301, Taiwan; 6 Institute for Translational Research in Biomedicine, Kaohsiung Chang Gung Memorial Hospital, Kaohsiung 83301, Taiwan

**Keywords:** WEE1, urothelial carcinoma, chemotherapy, DNA damage, cell cycle, *TP53* mutation

## Abstract

Urothelial carcinoma (UC) is characterized by a high incidence of *TP53* mutation, and overcoming resistance to cisplatin-based chemotherapy in UC is a major concern. Wee1 is a G2/M phase regulator that controls the DNA damage response to chemotherapy in *TP53*-mutant cancers. The combination of Wee1 blockade with cisplatin has shown synergistic efficacy in several types of cancers, but little is known regarding UC. The antitumor efficacy of the Wee1 inhibitor (AZD-1775) alone or in combination with cisplatin was evaluated in UC cell lines and a xenograft mouse model. AZD-1775 enhanced the anticancer activity of cisplatin by increasing cellular apoptosis. AZD-1775 inhibited the G2/M checkpoint, improving the sensitivity of mutant *TP53* UC cells to cisplatin by enhancing the DNA damage process. We confirmed that AZD-1775 combined with cisplatin reduced tumor volume and proliferation activity and increased the markers of cell apoptosis and DNA damage in the mouse xenograft model. In summary, the Wee1 inhibitor AZD-1775 combined with cisplatin elicited a promising anticancer efficacy in UC, and constitutes an innovative and promising therapeutic strategy.

## 1. Introduction

Metastatic urothelial carcinoma (mUC) is a lethal disease with a poor prognosis and 5-year survival rate barely exceeding 5% [1]. Platinum-based chemotherapy has been the standard of care in the first-line treatment of patients with mUC, with an objective response rate (ORR) of 40–50% and a median progression-free survival (PFS) of 7–8 months [2,3,4]. Once the disease progresses in patients on first-line platinum-based chemotherapy, the choice of second-line chemotherapy is limited because of the lack of effective therapeutic approaches [5]. Vinflunine, paclitaxel, and docetaxel are widely used in Europe and the U.S.; however, the PFS and overall survival (OS) are poor, at only 3.0 and 6.9 months, respectively [6,7]. Although recent advances in immune checkpoint inhibitors (ICIs) yield a robust survival benefit for mUC refractory to platinum-based chemotherapy, the ORRs of second-line ICIs were reported to be 13.4–21.1%, indicating that a majority of patients with mUC are unresponsive to the ICIs and required urgent therapeutic breakthrough [8,9,10]. The poor survival outcome is a result of the emerging and unavoidable resistance to chemotherapy [11,12]. Hence, effective therapies that are not susceptible to chemoresistance are urgently required for patients with mUC. 

*TP53* is a well-known tumor suppressor gene that is involved in several biological activities including the cell cycle, apoptosis, transcription, and DNA damage [13,14,15,16]. *TP53* mutation is the most common genetic alteration in human cancers, including UC of the bladder (UCB) and upper tract UC (UTUC) [17,18,19]. DNA damage activates cell cycle regulators and arrests the cell cycle in the G1/S and G2/M phases, enabling the action of several intricate mechanisms underlying DNA repair. Cancer cells lacking adequate p53 function depend on the G2/M cell cycle arrest in response to DNA damage [20,21]. Therefore, the G2/M checkpoint is crucial for the DNA repair process; a novel treatment for p53 dysregulated cancer cells aims to inhibit the G2/M checkpoint and cause G2/M arrest. 

Wee1 is a tyrosine kinase that regulates the G2/M checkpoint by inhibiting cyclin-dependent kinase 1 (CDK1), preventing entry into the mitosis phase in response to DNA damage [22,23]. Inhibition of Wee1 also triggers the phosphorylation of cyclin-dependent kinase 2 (CDK2), which subsequently enhances its activity, leading to abnormal DNA replication and causing double-stranded DNA breaks [24]. High Wee1 expression was reported to be strongly associated with increased cancer metastasis and poor survival in several cancer types, including breast cancer, ovarian cancer, colorectal cancer and laryngeal cancer [25,26,27,28]. Moreover, the silencing of Wee1 expression increases cell death, decreases metastasis, and sensitizes cancer cells to chemotherapy [29,30]. AZD-1775 (formerly MK-1775) is a potent ATP-competitive inhibitor of Wee1 kinase. Several preclinical studies have demonstrated the synergistic antitumor activity of AZD-1775 with chemotherapy in p53-deficient cancer cells [31,32,33]. Because UC is characterized by a high incidence of *TP53* mutation and often responds to cisplatin (CDDP) treatment, Wee1 blockade is expected to be synergistic with cisplatin for UC. However, few studies have demonstrated such a synergy in mUC. 

Therefore, we conducted this preclinical study to investigate the potential synergistic antitumor effect by combining cisplatin and AZD-1775 in p53-wild-type and p53-deficient UC cell lines. We found that AZD-1775 augmented the cytotoxic effect of cisplatin by increasing cell apoptosis through the DNA damage pathway. Our findings suggest that AZD-1775 in combination with chemotherapy may improve clinical outcomes for UC patients with *TP53* mutation. 

## 2. Materials and Methods

### 2.1. Cell Culture and Reagent Treatment

Human UC cell lines (BFTC-909, T24, J82, and RT4) (BCRC, Hsinchu, Taiwan) were used in this study. BFTC-909 cells were derived from patients with renal pelvis UC. T24, J84, and RT4 cells were human bladder cancer cells. BFTC-909 and J82 cells were cultured in Dulbecco’s modified Eagle medium, supplemented with 10% fetal bovine serum (FBS), 100 U/mL penicillin, and 100 μg/mL streptomycin. T24 and RT4 cells were cultured in McCoy’s 5A medium, which contained 10% FBS, 100 U/mL penicillin, and 100 μg/mL streptomycin. All cell lines were incubated at 37 °C with 5% CO_2_. Before undergoing analysis by apoptosis and cell cycle assays, cells were treated with a Wee1 inhibitor, 50 nM AZD-1775 (Selleckchem, Houston, TX, USA), 2 μM cisplatin (CDDP, Sigma-Aldrich, St. Louis, MO, USA), or a combination of both, for 48 h.

### 2.2. MTT Assay

Human urothelial cancer cell lines were seeded at a density of 2.5 *×* 10^3^ cells per well in 96-well plates, and allowed to attach overnight. After treatment with various experimental conditions for 72 h, 50 μL of MTT (3-(4,5-dimethylthiazol-2-yl)-2,5-diphenyltetrazolium bromide) (Sigma, St. Louis, MO, USA) was added to each well and incubated for 4 h. The supernatant was discarded and 50 μL of dimethyl sulfoxide (DMSO) (Sigma, St. Louis, MO, USA) was added to dissolve the formazan. The optical density at 570 nm was measured.

### 2.3. Apoptosis Assay

BFTC-909 and T24 cells were seeded into 6-well plates and allowed to attach overnight. The cells were harvested after the treatment with cisplatin alone, AZD-1775 alone, or cisplatin + AZD-1775 and stained with propidium iodide (PI) and Annexin V-APC (BioLegend, San Diego, CA, USA) for 15 min. Apoptotic cells were analyzed using BD FACSAria, and the results were analyzed using the FACSDIVA V8.0.2 software.

### 2.4. Cell Cycle Assay

BFTC-909 and T24 cells were seeded into 6-well plates and allowed to attach overnight. After the treatment with cisplatin alone, AZD-1775 alone, or cisplatin + AZD-1775, the cells were washed in phosphate-buffered saline (PBS), centrifuged, and fixed in cold 70% ethanol for 30 min at 4 °C. After being treated with ribonuclease A to remove RNA, the cells were stained with PI and incubated. Cell cycle distribution was analyzed using BD FACSAria, and results were analyzed using the FlowJo V10 software.

### 2.5. Colony Formation Assay

BFTC-909 and T24 cells were treated with cisplatin alone, AZD-1775 alone, or cisplatin + AZD-1775, for 24 h. A total of 100 cells/well were then seeded into 6-well plates and incubated for 14 days. The colonies were fixed and stained with 0.1% crystal violet for 20 min at room temperature. Colonies consisting of more than 50 cells were counted.

### 2.6. Western Blotting

After the human UC cells had been treated with different experimental conditions, cell lysates were harvested and lysed in a radioimmunoprecipitation assay (RIPA) buffer containing a protease inhibitor. The lysates were separated using sodium dodecyl sulfate-polyacrylamide gel electrophoresis (SDS-PAGE), transferred onto a polyvinylidene fluoride (PVDF) membrane (Amersham, UK), and blotted with primary antibodies followed by horseradish peroxidase (HRP)-conjugated secondary antibodies (BioCan Technologies, Vancouver, BC, Canada). The primary antibodies used included PARP (#9542, Cell Signaling, Danvers, MA, USA), Caspase3 (#9662, Cell Signaling, Danvers, MA, USA), cleaved-Caspase3 (#9664, Cell Signaling, Danvers, MA, USA), Caspase9 (#9508, Cell Signaling, Danvers, MA, USA), ATM (#2873, Cell Signaling, Danvers, MA, USA), p-ATM (ab81292, abcam, Cambridge, UK), γH2AX (#9718, Cell Signaling, Danvers, MA, USA), p-ATR (#30632, Cell Signaling, Danvers, MA, USA), ATR (GTX128146, Genetex, Irvine, CA, USA), p-Cyclin B1 (Invitrogen, Carlsbad, CA, USA), Cyclin B1 (MA5-14327, Invitrogen, Carlsbad, CA, USA), p-CDK1 (MA5-15062, Invitrogen, Carlsbad, CA, USA), CDK1 (33-1800, Invitrogen, Carlsbad, CA, USA), p-Chk1 (PA5-34625, Invitrogen, Carlsbad, CA, USA), Chk1 (MA1-23336, Invitrogen, Carlsbad, CA, USA), p-Chk2 (#2197, Cell Signaling, Danvers, MA, USA), Chk2 (GTX113055, Genetex, Irvine, CA, USA), p-Histone H3 (#3377, Cell Signaling, Danvers, MA, USA), Histone H3 (#9715, Cell Signaling, Danvers, MA, USA), p-CDK2 (ab176312, abcam, UK), CDK2 (MA5-17052, Invitrogen, Carlsbad, CA, USA), cyclin E (SC-247, Santa Cruz Biotechnology, Santa Cruz, CA, USA) and β-actin (MA5-11869, I Invitrogen, Carlsbad, CA, USA). Protein bands were detected with enhanced chemiluminescence (ECL) reagent (Thermo Fisher Scientific, Inc., Waltham, MA, USA), and the relative protein expression levels were quantified using ImageJ software. 

### 2.7. Immunofluorescence Staining

Cisplatin-treated, AZD-1775-treated, and combination cisplatin with AZD-1775-treated UC cells were fixed with 4% paraformaldehyde for 15 min, permeabilized with 0.3% Triton X-100 in PBS, blocked with 3% bovine serum albumin in PBS and incubated with anti-γH2AX at 4 °C overnight. The staining was completed with Alexa Fluor 594-labeled anti-rabbit IgG (Thermo Fisher Scientific, Inc., Waltham, MA, USA) for 1 h. Cell nuclei were stained with 4′,6-diamidino-2-phenylindole (DAPI) for 15 min. The slides were observed using fluorescence microscopy. 

### 2.8. Comet Assay

The Comet assay kit (Trevigen, Minneapolis, MN, USA) was used according to the manufacturer’s instructions to assess the DNA damage level. Harvested cells were suspended in low-melting agarose and spread on a comet slide. The slides were incubated at 41 °C for 10 min to enhance the adhesion and gel polymerization. The slides were then immersed in the lysis buffer at 4 °C, then in an alkaline solution (0.3 M NaOH, 1 mM EDTA) and neutralized in TBE buffer. Electrophoresis was conducted in the same TBE buffer at 1 V/cm for 10 min. The slides were fixed with cold 70% methanol, air dried, and stained with SYBR-Red (Molecular Probes, Carlsbad, CA, USA). Images were captured by fluorescence microscopy.

### 2.9. Animal Model

The animal study was approved by the Institutional Animal Care and Use Committee of Kaohsiung Chang Gung Memorial Hospital and was conducted following the Animal Research: Reporting in Vivo Experiments (ARRIVE) guidelines. Seven-week-old NOD-SCID mice from the National Laboratory Animal Center of Taiwan were injected with 3 × 10^6^ BFTC-909 cells. When tumor volumes reached 50–100 mm^3^, the mice were randomly divided into four groups (control, cisplatin alone, AZD-1775 alone, and cisplatin + AZD-1775), with each group consisting of five mice. The mice were then injected with different reagents for a period of 2 weeks. Tumor volumes were calculated using the formula (width^2^ × length)/2.

### 2.10. Immunohistochemical (IHC) Staining

Tumors sampled from the xenograft mouse were fixed in 10% formalin, dehydrated, embedded in paraffin, and cut into 4 μm sections. The sections were deparaffinized with xylene and hydrated using a series of graded ethanol solutions. The sections were subjected to antigen retrieval, using a retrieval solution. Hematoxylin and eosin staining kits (Abcam, Cambridge, UK) were used, according to the manufacturer’s instructions. The sections were then stained with primary antibodies against Ki67 and γH2AX at 4 °C overnight. The sections were incubated with HRP-conjugated secondary antibodies at 37 °C, and 3,3′-diaminobenzidine tetrachloride (DAB) was used to detect the HRP activity.

### 2.11. Statistical Analysis

All experiments were conducted in triplicate. Data are expressed as the mean ± standard error. Differences between experimental groups were determined using one-way analysis of variance and Tukey’s post hoc test for multiple comparisons. An independent *t* test was used to compare the differences between two groups. *p* < 0.05 indicated statistical significance.

## 3. Results

### 3.1. Combining AZD-1775 and Cisplatin Synergistically Reduces the Viability of UC Cells

**Figure 1:**
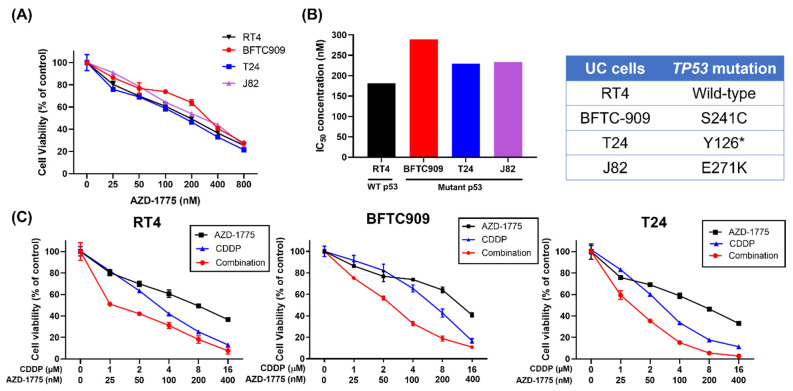
AZD−1775 alone or in combination with cisplatin inhibits growth in UC cells. (**A**) Cell viability was measured by MTT assay in UC cells (BFTC-909, T24, J82, and RT4) treated with increasing doses of AZD−1775. (**B**) Correlation of AZD−1775 IC_50_ values with *TP53* status in UC cells. Y126* indicated substitution of the amino acid tyrosine (Y) with a premature stop codon (*) at position 126. (**C**) Analysis of cell viability in UC cells treated with AZD−1775 + cisplatin (CDDP).

To examine the antitumor effect of AZD-1775 on UC cells, we treated four UC cell lines (RT4, BFTC-909, T24, and J82) with varying doses of AZD-1775, from 25 to 800 nM, and analyzed cell viability with an MTT assay. As shown in Figure 1A, a low AZD-1775 concentration was associated with minimal cytotoxicity, whereas an increased concentration of 100 nM resulted in approximately 40% cell death in all the UC cell lines except BFTC-909. Additionally, cell death increased with AZD-1775 dose across all UC cell lines. We further compared the IC_50_ of AZD-1775 between wild-type p53 cells (RT4) and mutant p53 cells (BFTC-909, T24, and J82). Our results showed that the IC_50_ in the p53 mutant UC cells was approximately 170–230 nM, which was higher than that in the p53 wild-type cells (Figure 1B). We treated UC cells with cisplatin (CDDP)+ AZD-1775 to verify the synergistic effect of inhibiting Wee1. Cisplatin (2 μΜ) alone resulted in cell death of about 20%, whereas the combination with 50 nM AZD-1775 resulted in cell death of 40% in the BFTC-909 cells. The synergistic effect of cisplatin with AZD-1775 was also observed in T24 cells. AZD-1775 also enhanced the cytotoxicity of cisplatin in wild-type p53 (RT4) cells (Figure 1C).

### 3.2. Concomitant Treatment of AZD-1775 with Cisplatin Reduced Proliferation and Enhanced Apoptosis of UC Cells

**Figure 2:**
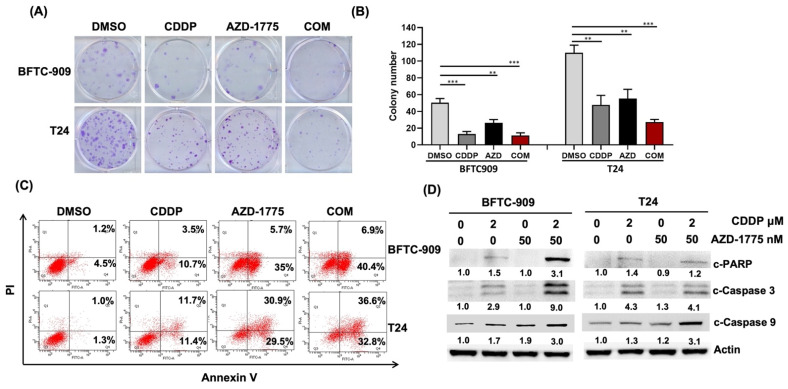
Combination treatment of cisplatin and AZD−1775 synergistically reduces cell proliferation and enhances apoptosis in UC cells. (**A**) Colony formation assay for *TP53*-mutant UC cells (BFTC-909 and T24) after treatment with cisplatin alone, AZD−1775 alone, or cisplatin + AZD−1775. (**B**) Quantitative results of colony formation assay. (** *p* < 0.01, *** *p* < 0.001). (**C**) Apoptotic cells were analyzed using Annexin V-APC/propidium iodide (PI) apoptosis assay. (**D**) Western blot was used to examine the protein expression of apoptosis-related proteins in UC cells.

Our earlier findings demonstrated that AZD-1775 increased the cytotoxicity of cisplatin in UC cells. We further investigated the effect of combining AZD-1775 and cisplatin on cell proliferation. Based on colony analysis, cisplatin alone markedly reduced the number of colonies in T24 and BFTC-909 cells. In addition, AZD-1775 enhanced cisplatin-induced inhibition of cellular proliferation (Figure 2A,B). We used Annexin V staining to verify the effect of AZD-1775 on cisplatin-induced antiproliferative activity. As expected, the proportion of Annexin V^+^ cells in the cisplatin + AZD-1775 group was higher than in the cisplatin-alone group in both BFTC-909 and T24 cells (Figure 2C). Because cisplatin and the Wee1 inhibitor had antitumor effects through the induction of apoptosis, we also measured the expression of apoptotic proteins, specifically PARP, Caspase-3, and Caspase-9. Cisplatin alone led to cytotoxic cell death by increasing the relevant apoptotic process. As shown in Figure 2D, apoptotic proteins increased in the cisplatin-alone group and increased the most in the cisplatin + AZD-1775 group. This finding suggests that the combination of cisplatin and Wee1 inhibitor enhanced the antitumor ability by inducing apoptosis in the UC cells.

### 3.3. AZD-1775 Reverses the Cisplatin-Induced G2/M Phase Cell Cycle Arrest in UC Cells

**Figure 3:**
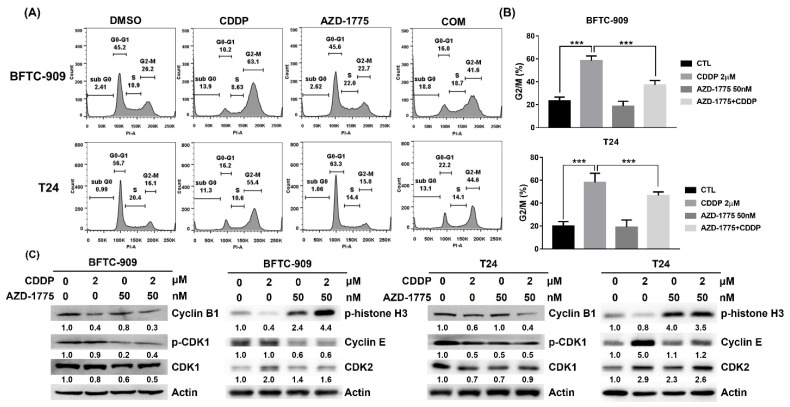
AZD−1775 markedly reverses the cisplatin-induced G2/M phase cell cycle arrest in UC cells. (**A**) Cell cycle distribution of BFTC-909 and T24 cells after treatments were analyzed by flow cytometry using PI staining. (**B**) Bar charts representing the percentage of cells in each phase of the cell cycle (*** *p* < 0.001). (**C**) Western blot was used to examine the protein expression of G2/M checkpoint regulators in UC cells.

Wee1 is a crucial component of the G2/M checkpoint that prevents entry into mitosis in response to cell DNA damage. Thus, we examined whether the chemosensitizing effects of AZD-1775 were related to interference from cisplatin-induced G2/M phase arrest. AZD-1755 markedly reversed the accumulation of G2/M phase cells when BFTC-909 and T24 cells were treated with AZD-1775 before cisplatin treatment (Figure 3A,B). Wee1 arrests the G2/M checkpoint in response to DNA damage by phosphorylating CDK1 and cyclin B1 complex at the tyrosine 15 residue. According to our findings, the phosphorylation level of CDK1 decreased in both the AZD-1775 alone and the cisplatin + AZD-1775 groups (Figure 3C). We also verified the expression of cyclin B1, which binds to phosphorylated CDK1 in the regulation of G2/M phase progression. The expression of cyclin B1 exhibited a large decrease when the UC cells were treated with AZD-1775 + cisplatin, indicating that more UC cells enter the mitotic phase and do not have sufficient time to repair DNA damage. The same results were observed in two other G2/M markers, cyclin E and CDK2 (Figure 3C). Furthermore, the M phase marker p-Histone H3 was elevated when cells were treated with AZD-1775 alone or in combination with CDDP (Figure 3C). These findings jointly indicate that AZD-1775 enhances the progression of the cell cycle in UC cells with p53 dysfunction when acting in response to DNA damage, and improves the antitumor effects of cisplatin.

### 3.4. AZD-1775 Enhances Cisplatin-Induced DNA Damage

**Figure 4:**
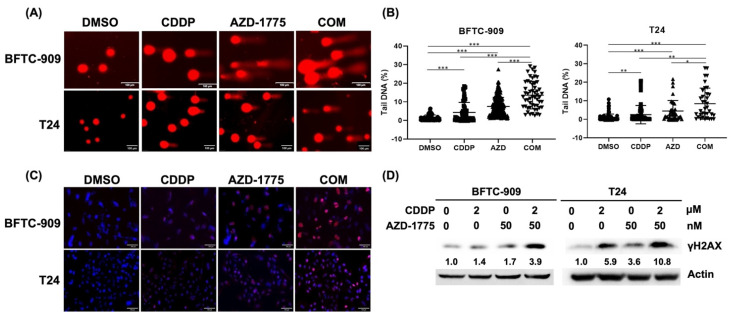
Combination treatment with cisplatin and AZD−1775 enhances DNA damage in BFTC-909 and T24 cells. (**A**) DNA damage was visualized under a fluorescence microscope. Scale bar, 100 μm. (**B**) Tail DNA was quantified using the OpenComet software. (* *p* < 0.05, ** *p* < 0.01, *** *p* < 0.001). (**C**,**D**) Phosphorylation of H2AX (γH2AX) was detected by immunofluorescence and Western blot. Scale bar, 100 μm.

Cisplatin is an alkylating agent that binds to DNA and damages cancer cells. We used the comet assay (single-cell gel electrophoresis) as a simple and sensitive method for DNA strand break detection in eukaryotic cells to determine whether AZD-1775 is synergistic with cisplatin. The percentage of tail DNA in the cisplatin + AZD-1775 group was markedly higher than in the control and cisplatin-alone groups in the BFTC-909 and T24 cells, suggesting that AZD-1775 with cisplatin induces greater DNA breakdown (Figure 4A,B). We also detected a marker for DNA damage, the phosphorylated expression of H2AX (γH2AX) in BFTC-909 and T24 cells. UC cells treated with AZD-1775 plus cisplatin markedly increased the fluorescent intensity of γH2AX and enhanced the protein expression in the Western blotting assay (Figure 4C,D). These findings suggest that the Wee1 inhibitor AZD-1775 enhances cisplatin-induced DNA damage in UC cells. 

### 3.5. AZD-1775 Interferes with the Expression of DNA Repair-Related Proteins

**Figure 5:**
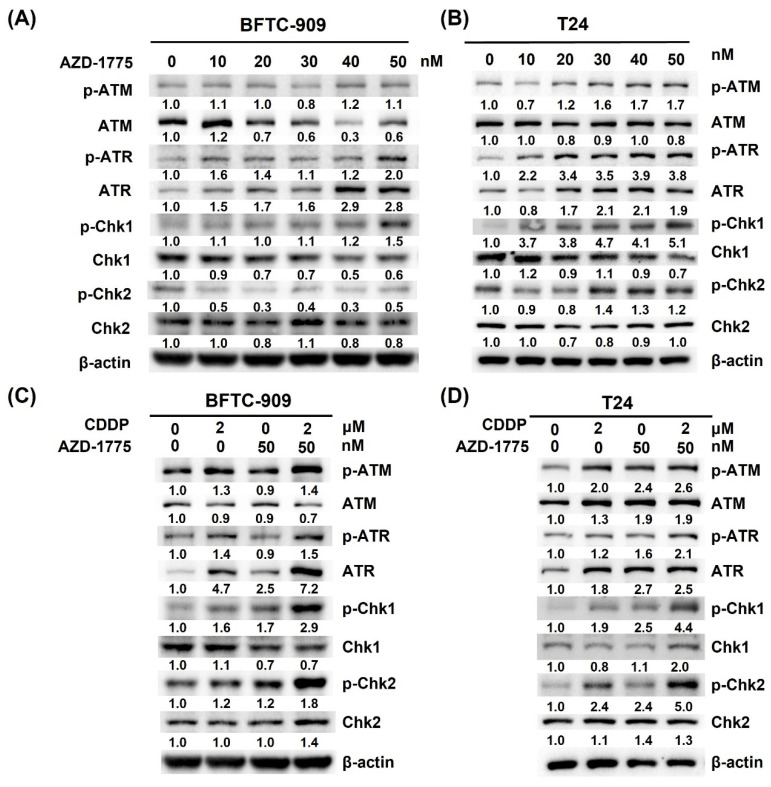
AZD−1775 alone or in combination with cisplatin upregulated DNA repair-related protein expression in BFTC-909 and T24 cells. (**A**,**B**) Dose-dependent effect of AZD−1775 in BFTC-909 and T24 cells. (**C**,**D**) Combination of cisplatin and AZD-1775 in BFTC-909 and T24 cells.

Because AZD-1775 enhances cisplatin-induced DNA damage, and cisplatin-induced double-strand breaks could be repaired by homologous recombination (HR), we further investigated whether AZD-1775 alone or in combination with cisplatin could lead to a defect in HR-mediated DNA damage response. Immunoblots of cell lysates showed that administration of AZD-1775 increased the phosphorylation of Chk1 and ATR in BFTC909 and T24 cells in a dose-dependent manner. However, p-ATM and p-Chk2 were expressed in T24 cells but not in BFTC-909 cells (Figure 5A,B). These data indicate that AZD-1775 treatment upregulated the DNA damage response pathway (ATR-Chk1 and ATM-Chk2). When combined with cisplatin, p-ATR, p-Chk2, p-ATM and p-Chk1 increased in the BFTC-909 and T24 cells (Figure 5C,D). These findings suggest that Wee1 inhibition leads to the activation of the DNA damage response pathway (ATR and ATM), which prompted us to use the combination of ATR or ATM with Wee1 inhibition to enhance the cytotoxicity of urothelial carcinoma.

### 3.6. AZD-1775 Increases the Sensitivity of UC Cells to Cisplatin in Mouse Tumor Xenografts

**Figure 6:**
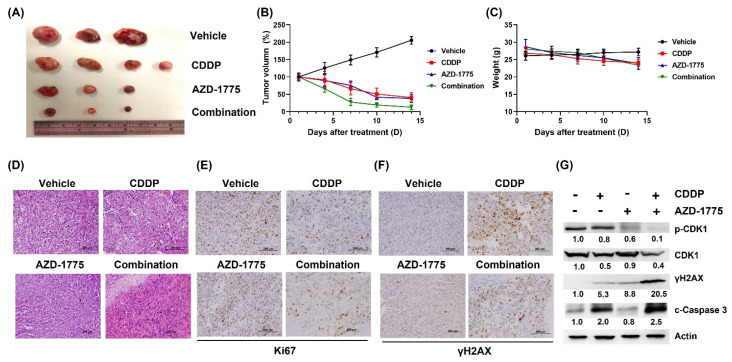
Concomitant treatment with AZD−1775 and cisplatin enhances antitumor efficacy and induces apoptosis in BFTC-909 cells in a mouse xenograft model. (**A**) Representative images of the solid tumors obtained from each group. (**B**) Tumor volume measured during the treatment periods in each group. (**C**) Body weights of the mice monitored during the treatment periods. (**D**) Hematoxylin and eosin (H&E) staining of tumor samples. Scale bar, 200 μm. (**E**,**F**) Immunohistochemical staining of Ki67 and γ-H2AX in tumor samples. Scale bar, 200 μm. (**G**) Mechanism validation confirmed by Western blot.

We used NOD-SCID xenograft mice to verify the efficacy of AZD-1775 + cisplatin observed in vitro. Monotherapy with cisplatin or AZD-1775 showed a modest antitumor effect, but cisplatin + AZD-1775 produced the greatest inhibition of tumor growth (Figure 6A,B). Notably, no significant differences in body weight were observed between the experimental groups (Figure 6C). H&E staining results of excised tumors showed a significant reduction in tumor size with prominent necrotic and fibrotic surrounding stroma in the cisplatin + AZD-1775 group (Figure 6D). IHC staining results showed that the proliferation indicator Ki-67 decreased in the combination therapy group (Figure 6E,F). At the molecular level, AZD-1775 + cisplatin induced apoptosis (c-caspase 3), DNA damage (γH2AX), and decreased levels of G2/M regulator (pCDK1) in xenograft mice (Figure 6G). In conclusion, AZD-1775 enhanced the antitumor efficacy of cisplatin by disrupting G2/M cell cycle arrest and increasing DNA damage.

## 4. Discussion

Cisplatin (CDDP)-based chemotherapy has long been the first-line treatment for mUC. Although mUC is sensitive to cisplatin-based chemotherapy, the duration of chemotherapy treatment may not last long, because of the inevitable emergence of drug resistance. Consequently, researchers have investigated cisplatin resistance and the synergy between cisplatin and new therapeutic agents. The inhibition of Wee1 kinase has been demonstrated to overcome resistance to cisplatin associated with several solid tumors harboring TP53 mutations [34,35,36,37]. We demonstrated that the Wee1 inhibitor AZD-1775 enhances the anticancer efficacy of cisplatin in UC cells, facilitating the progression of the G2/M phase, and leading to an increase in DNA strand breaks and cell apoptosis, both in vitro and in vivo. 

Extensive research has been conducted into the targeting of Wee1 kinase, a crucial regulator of the G2/M checkpoint, as a therapeutic strategy for treating *TP53* mutant cancer. However, the efficacy of Wee1 inhibition is not restricted to *TP53* mutant cancer cells. Our study revealed that AZD-1775 monotherapy resulted in a dose-dependent death of *TP53* wide-type and mutant UC cells in vitro (Figure 1). Irrespective of the presence of *TP53* mutations, the combination of AZD-1775 and cisplatin showed synergistic cytotoxicity in all UC cells. The aforementioned outcome has been observed not only by our team, but also by other researchers. Murakami and colleagues demonstrated that the administration of AZD-1775 alone effectively inhibited the growth of UC cell lines in a dose-dependent manner regardless of TP53 status, which aligns with our own experimental observations. However, in contrast to Murakami’s findings, our data revealed a synergistic effect when AZD-1775 was combined with other treatments, irrespective of TP53 status. This disparity could potentially be attributed to variations in drug concentrations and treatment durations employed in the respective studies [38]. Furthermore, the administration of AZD-1775 leads to wide-type *TP53* tumor shrinkage through the PDX model and cancer organoid spheroids [38]. Not only UC, but also AZD-1775, exhibit a robust cytotoxicity against both p53-mutant or p53 wild type pancreatic adenocarcinoma and ovarian cancer [39,40]. A recent phase I trial of AZD-1775 monotherapy demonstrated a favorable ORR (14%) in advanced solid cancers [41]. Notably, one patient who had neither *TP53* nor *BRCA1/2* mutation achieved a partial response to AZD-1775, indicating that the antitumor effect of AZD-1775 was not limited to *TP53* mutant tumors. These effects may be related to the fact that AZD-1775 induces DNA double-strand breakdown in the S phase, and just functions as a G2/M phase regulator. Further in-depth studies should be conducted to investigate the antitumor mechanism of the Wee1 inhibitor and to explore the potential predictive markers. 

The combination of Wee1 inhibitor with chemotherapy has exhibited synergistic cytotoxicity in several types of mutant *TP53* cancers, including head and neck cancer, osteosarcoma, breast cancer, and ovarian cancer [34,35,36,37]. In this study, we report that AZD-1775 with cisplatin exhibited enhanced toxicity to UC cells by increasing DNA damage and impairing cell recovery. AZD-1775 facilitated the progression of UC cells to the G2/M phase, increased cell apoptosis, and increased susceptibility to cisplatin-related DNA damage. In the animal model, the combination of AZD-1775 and cisplatin had the best effect on tumor shrinkage. These results are clinically significant, because cisplatin-based chemotherapy remains the gold standard for the first-line treatment of mUC. Although ICIs have been widely used for mUC, the responsiveness to cisplatin-based first-line chemotherapy remains the primary determinant in choosing a candidate for maintenance immunotherapy [42]. Notably, Wee1 inhibition leads to increased eradication of tumor cells by cytotoxic T-lymphocyte through tumor necrosis factor (TNF)-α-dependent cell death in several cancer cells [43]. The blockade of Wee1 kinase also enhances natural-killer-cell-mediated granzyme-B-dependent cell lysis in head and neck cancer [44]. A recent study by Taniguchi et al. demonstrated that Wee1 inhibition promotes CD8^+^ cytotoxic T cell activation and increases PD-L1 expression through STING and STAT1 pathways in small-cell lung cancer [45]. Combined AZD-1775 and anti-PD-L1 treatment resulted in a marked reduction in tumor volume in genetically engineered mouse models. Concomitant treatment with a Wee1 inhibitor and an ICI may be a potential therapeutic strategy for the successful treatment of mUC because UC is considered an inflamed and immune-responsive tumor.

Mechanistically, the Wee1 inhibitor AZD-1775 induced DNA breaks and apoptosis in UC cells. The subsequent result of Wee1 inhibition leads to activation of the ATR-Chk1 and ATM-Chk2 signaling pathways, suggesting that activation of the HR signaling pathway may be due to the resistance of Wee1 inhibition in UC cells. Blocking the activity of Wee1 can cause replication stress and DNA damage, which in turn triggers the activation of the ATR and ATM pathways. Inhibition of Wee1 reduces the phosphorylation of CDK1, leading to the activation of CDK1 and inappropriate initiation of DNA synthesis, resulting in replication stress. ATR and ATM are then activated in response to this replication stress, initiating downstream signaling cascades that include the activation of Chk1 or Chk2. In a recent investigation by Rødland and colleagues, administration of a Wee1 inhibitor led to augmented phosphorylation of Chk1 S317 and ATR expression in all lung cancer cells [46]. Our findings were in agreement with these results, indicating that the activation of ATR may play a role in repressing the induction of DNA damage following inhibition of Wee1. Earlier research has demonstrated that in small-cell lung cancer, the activation of Chk1 through the upregulation of AXL and MET pathways is a significant mechanism of resistance to AZD-1775 [47]. Thus, combining ATR with Chk1 inhibition can be a rational approach to overcoming resistance to Wee1 inhibition, and it has demonstrated a synergistic anti-tumor effect in multiple types of cancer cells [48,49,50]. While the application of ATR inhibitors in urothelial carcinoma is still in its early stages of research, a potential new therapeutic approach involves combining Wee1 and ATR inhibition with conventional platinum-based chemotherapy. This approach is believed to hold promise, as preclinical studies have indicated that it could enhance the effectiveness of chemotherapy by increasing DNA damage and cell death in cancer cells. Further clinical trials are needed to confirm these findings, but they offer a hopeful avenue for the development of more potent treatments for urothelial carcinoma [51].

## 5. Conclusions

**Figure 7:**
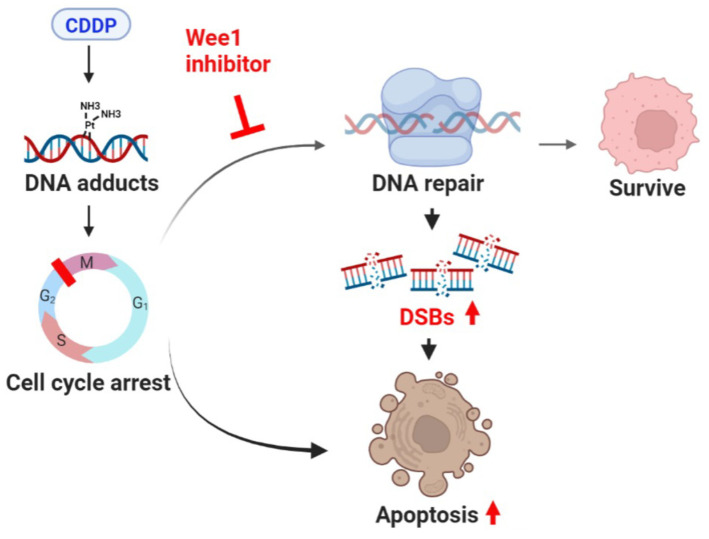
Illustration of the mechanism of AZD−1775 enhancing cytotoxicity of cisplatin. Figure was created with BioRender.com (accessed on 9 August 2022).

Our results demonstrated that AZD-1775 markedly improved the anticancer efficacy of cisplatin in UC cells, resulting in increased cell apoptosis, DNA damage, and decreased DNA repair mechanism (Figure 7).

## Data Availability

Not applicable.

## References

[1] Siegel R.L., Miller K.D., Goding Sauer A., Fedewa S.A., Butterly L.F., Anderson J.C., Cercek A., Smith R.A., Jemal A. (2020). Cancer statistics, 2020. CA Cancer J. Clin..

[2] Von Der Maase H., Hansen S.W., Roberts J.T., Dogliotti L., Oliver T., Moore M.J., Bodrogi I., Albers P., Knuth A., Lippert C.M. (2000). Gemcitabine and Cisplatin Versus Methotrexate, Vinblastine, Doxorubicin, and Cisplatin in Advanced or Metastatic Bladder Cancer: Results of a Large, Randomized, Multinational, Multicenter, Phase III Study. J. Clin. Oncol..

[3] von der Maase H., Sengelov L., Roberts J.T., Ricci S., Dogliotti L., Oliver T., Moore M.J., Zimmermann A., Arning M. (2005). Long-term survival results of a randomized trial comparing gemcitabine plus cisplatin, with methotrexate, vinblastine, doxorubicin, plus cisplatin in patients with bladder cancer. J. Clin. Oncol..

[4] Bellmunt J., von der Maase H., Mead G.M., Skoneczna I., De Santis M., Daugaard G., Boehle A., Chevreau C., Paz-Ares L., de Wit R. (2012). Randomized phase III study comparing paclitaxel/cisplatin/gemcitabine and gemcitabine/cisplatin in patients with locally advanced or metastatic urothelial cancer without prior systemic therapy: EORTC Intergroup Study 30987. J. Clin. Oncol..

[5] El Rassy E., Assi T., Bakouny Z., Pavlidis N., Kattan J. (2019). Beyond first line systemic treatment for metastatic urothelial carcinoma of the bladder. Clin. Transl. Oncol..

[6] Bellmunt J., Théodore C., Demkov T., Komyakov B., Sengelov L., Daugaard G., Caty A., Carles J., Jagiello-Gruszfeld A., von der Maase H. (2009). Phase III trial of vinflunine plus best supportive care compared with best supportive care alone after a platinum-containing regimen in patients with advanced transitional cell carcinoma of the urothelial tract. J. Clin. Oncol..

[7] Vaughn D.J., Broome C.M., Hussain M., Gutheil J.C., Markowitz A.B. (2002). Phase II Trial of Weekly Paclitaxel in Patients with Previously Treated Advanced Urothelial Cancer. J. Clin. Oncol..

[8] Rosenberg J.E., Hoffman-Censits J., Powles T., Van Der Heijden M.S., Balar A.V., Necchi A., Dawson N., Balmanoukian A., Loriot Y., Dreicer R. (2016). Atezolizumab in patients with locally advanced and metastatic urothelial carcinoma who have progressed following treatment with platinum-based chemotherapy: A single-arm, multicentre, phase 2 trial. Lancet.

[9] Bellmunt J., De Wit R., Vaughn D.J., Fradet Y., Lee J.L., Fong L., Vogelzang N.J., Climent M.A., Petrylak D.P., Bajorin D.F. (2017). Pembrolizumab as Second-Line Therapy for Advanced Urothelial Carcinoma. N. Engl. J. Med..

[10] Powles T., Durán I., Van der Heijden M.S., Loriot Y., Vogelzang N.J., De Giorgi U., Oudard S., Retz M., Castellano D., Ravaud A. (2018). Atezolizumab Versus Chemotherapy in Patients With Platinum-Treated Locally Advanced or Metastatic Urothelial Carcinoma (IMvigor211): A Multicentre, Open-Label, Phase 3 Randomised Controlled Trial. Lancet.

[11] Drayton R.M., Catto J.W. (2012). Molecular mechanisms of cisplatin resistance in bladder cancer. Expert Rev. Anticancer Ther..

[12] Vallo S., Michaelis M., Rothweiler F., Bartsch G., Gust K.M., Limbart D.M., Rödel F., Wezel F., Haferkamp A., Cinatl J. (2015). Drug-Resistant Urothelial Cancer Cell Lines Display Diverse Sensitivity Profiles to Potential Second-Line Therapeutics. Transl. Oncol..

[13] Chen J. (2016). The Cell-Cycle Arrest and Apoptotic Functions of p53 in Tumor Initiation and Progression. Cold Spring Harb. Perspect. Med..

[14] Fridman J.S., Lowe S.W. (2003). Control of apoptosis by p53. Oncogene.

[15] Beauséjour C.M., Krtolica A., Galimi F., Narita M., Lowe S.W., Yaswen P., Campisi J. (2003). Reversal of human cellular senescence: Roles of the p53 and p16 pathways. EMBO J..

[16] Meek D.W. (2009). Tumour suppression by p53: A role for the DNA damage response?. Nat. Rev. Cancer.

[17] Cancer Genome Atlas Research Network (2014). Comprehensive molecular characterization of urothelial bladder carcinoma. Nature.

[18] Robertson A.G., Kim J., Al-Ahmadie H., Bellmunt J., Guo G., Cherniack A.D., Hinoue T., Laird P.W., Hoadley K.A., Tam A. (2017). Comprehensive Molecular Characterization of Muscle-Invasive Bladder Cancer. Cell.

[19] Audenet F., Attalla K., Sfakianos J.P. (2018). The evolution of bladder cancer genomics: What have we learned and how can we use it?. Urologic Oncology: Seminars and Original Investigations.

[20] Benedict B., van Harn T., Dekker M., Hermsen S., Kucukosmanoglu A., Pieters W., Delzenne-Goette E., Dorsman J.C., Petermann E., Te Riele H. (2018). Loss of P53 Suppresses Replication-Stress-Induced DNA Breakage in G1/S Checkpoint Deficient Cells. eLife.

[21] Hafner A., Bulyk M.L., Jambhekar A., Lahav G. (2019). The multiple mechanisms that regulate p53 activity and cell fate. Nat. Rev. Mol. Cell Biol..

[22] Matheson C.J., Backos D.S., Reigan P. (2016). Targeting WEE1 Kinase in Cancer. Trends Pharmacol. Sci..

[23] Do K., Doroshow J.H., Kummar S. (2013). Wee1 kinase as a target for cancer therapy. Cell Cycle.

[24] Moiseeva T.N., Qian C., Sugitani N., Osmanbeyoglu H.U., Bakkenist C.J. (2019). WEE1 kinase inhibitor AZD1775 induces CDK1 kinase-dependent origin firing in unperturbed G1- and S-phase cells. Proc. Natl. Acad. Sci. USA.

[25] de Nonneville A., Finetti P., Birnbaum D., Mamessier E., Bertucci F. (2021). WEE1 Dependency and Pejorative Prognostic Value in Triple-Negative Breast Cancer. Adv. Sci..

[26] Slipicevic A., Holth A., Hellesylt E., Tropé C.G., Davidson B., Flørenes V.A. (2014). Wee1 is a novel independent prognostic marker of poor survival in post-chemotherapy ovarian carcinoma effusions. Gynecol. Oncol..

[27] Ge X.-C., Wu F., Li W.-T., Zhu X.-J., Liu J.-W., Wang B.-L. (2017). Upregulation of WEE1 is a potential prognostic biomarker for patients with colorectal cancer. Oncol. Lett..

[28] Yuan M.-L., Li P., Xing Z.-H., Di J.-M., Liu H., Yang A.-K., Lin X.-J., Jiang Q.-W., Yang Y., Huang J.-R. (2018). Inhibition of WEE1 Suppresses the Tumor Growth in Laryngeal Squamous Cell Carcinoma. Front. Pharmacol..

[29] Geenen J.J., Schellens J.H. (2017). Molecular Pathways: Targeting the Protein Kinase Wee1 in Cancer. Clin. Cancer Res..

[30] Meng X., Bi J., Li Y., Yang S., Zhang Y., Li M., Liu H., Li Y., Mcdonald M.E., Thiel K.W. (2018). AZD1775 Increases Sensitivity to Olaparib and Gemcitabine in Cancer Cells with p53 Mutations. Cancers.

[31] Hirai H., Iwasawa Y., Okada M., Arai T., Nishibata T., Kobayashi M., Kimura T., Kaneko N., Ohtani J., Yamanaka K. (2009). Small-molecule inhibition of Wee1 kinase by MK-1775 selectively sensitizes p53-deficient tumor cells to DNA-damaging agents. Mol. Cancer Ther..

[32] Bridges K.A., Hirai H., Buser C.A., Brooks C., Liu H., Buchholz T.A., Molkentine J.M., Mason K.A., Meyn R.E. (2011). MK-1775, a Novel Wee1 Kinase Inhibitor, Radiosensitizes p53-Defective Human Tumor Cells. Clin. Cancer Res..

[33] Rajeshkumar N., De Oliveira E., Ottenhof N., Watters J., Brooks D., Demuth T., Shumway S.D., Mizuarai S., Hirai H., Maitra A. (2011). MK-1775, a Potent Wee1 Inhibitor, Synergizes with Gemcitabine to Achieve Tumor Regressions, Selectively in p53-Deficient Pancreatic Cancer Xenografts. Clin. Cancer Res..

[34] Zheng H., Shao F., Martin S., Xu X., Deng C.-X. (2017). WEE1 inhibition targets cell cycle checkpoints for triple negative breast cancers to overcome cisplatin resistance. Sci. Rep..

[35] Osman A.A., Monroe M.M., Alves M.V.O., Patel A.A., Katsonis P., Fitzgerald A.L., Neskey D.M., Frederick M.J., Woo S.H., Caulin C. (2015). Wee-1 Kinase Inhibition Overcomes Cisplatin Resistance Associated with High-Risk *TP53* Mutations in Head and Neck Cancer through Mitotic Arrest Followed by Senescence. Mol. Cancer Ther..

[36] Hu Z., Li L., Lan W., Wei X., Wen X., Wu P., Zhang X., Xi X., Li Y., Wu L. (2022). Enrichment of Wee1/CDC2 and NF-κB Signaling Pathway Constituents Mutually Contributes to CDDP Resistance in Human Osteosarcoma. Cancer Res. Treat..

[37] Li J., Pan C., Boese A.C., Kang J., Umano A.D., Magliocca K.R., Yang W., Zhang Y., Lonial S., Jin L. (2020). DGKA Provides Platinum Resistance in Ovarian Cancer Through Activation of c-JUN–WEE1 Signaling. Clin. Cancer Res..

[38] Murakami K., Kita Y., Sakatani T., Hamada A., Mizuno K., Nakamura K., Takada H., Matsumoto K., Sano T., Goto T. (2021). Antitumor effect of WEE1 blockade as monotherapy or in combination with cisplatin in urothelial cancer. Cancer Sci..

[39] Zhang M., Dominguez D., Chen S., Fan J., Qin L., Long A., Li X., Zhang Y., Shi H., Zhang B. (2017). WEE1 inhibition by MK1775 as a single-agent therapy inhibits ovarian cancer viability. Oncol. Lett..

[40] Hartman S.J., Bagby S.M., Yacob B.W., Simmons D.M., MacBeth M., Lieu C.H., Davis S.L., Leal A.D., Tentler J.J., Diamond J.R. (2021). WEE1 Inhibition in Combination With Targeted Agents and Standard Chemotherapy in Preclinical Models of Pancreatic Ductal Adenocarcinoma. Front. Oncol..

[41] Takebe N., Naqash A.R., Coyne G.O., Kummar S., Do K., Bruns A., Juwara L., Zlott J., Rubinstein L., Piekarz R. (2021). Safety, Antitumor Activity, and Biomarker Analysis in a Phase I Trial of the Once-daily Wee1 Inhibitor Adavosertib (AZD1775) in Patients with Advanced Solid Tumors. Clin. Cancer Res..

[42] Powles T., Park S.H., Voog E., Caserta C., Valderrama B.P., Gurney H., Kalofonos H., Radulović S., Demey W., Ullén A. (2020). Avelumab Maintenance Therapy for Advanced or Metastatic Urothelial Carcinoma. N. Engl. J. Med..

[43] Sun L., Moore E., Berman R., Clavijo P.E., Saleh A., Chen Z., Van Waes C., Davies J., Friedman J., Allen C.T. (2018). WEE1 kinase inhibition reverses G2/M cell cycle checkpoint activation to sensitize cancer cells to immunotherapy. Oncoimmunology.

[44] Friedman J., Morisada M., Sun L., Moore E.C., Padget M., Hodge J.W., Schlom J., Gameiro S., Allen C.T. (2018). Inhibition of WEE1 kinase and cell cycle checkpoint activation sensitizes head and neck cancers to natural killer cell therapies. J. Immunother. Cancer.

[45] Taniguchi H., Caeser R., Chavan S.S., Zhan Y.A., Chow A., Manoj P., Uddin F., Kitai H., Qu R., Hayatt O. (2022). WEE1 inhibition enhances the antitumor immune response to PD-L1 blockade by the concomitant activation of STING and STAT1 pathways in SCLC. Cell Rep..

[46] Rødland G.E., Hauge S., Hasvold G., Bay L.T.E., Raabe T.T.H., Joel M., Syljuåsen R.G. (2021). Differential Effects of Combined ATR/WEE1 Inhibition in Cancer Cells. Cancers.

[47] Sen T., Tong P., Diao L., Li L., Fan Y., Hoff J., Heymach J.V., Wang J., Byers L.A. (2017). Targeting AXL and mTOR Pathway Overcomes Primary and Acquired Resistance to WEE1 Inhibition in Small-Cell Lung Cancer. Clin. Cancer Res..

[48] Bukhari A.B., Lewis C.W., Pearce J.J., Luong D., Chan G.K., Gamper A.M. (2019). Inhibiting Wee1 and ATR kinases produces tumor-selective synthetic lethality and suppresses metastasis. J. Clin. Investig..

[49] Jin J., Fang H., Yang F., Ji W., Guan N., Sun Z., Shi Y., Zhou G., Guan X. (2018). Combined Inhibition of ATR and WEE1 as a Novel Therapeutic Strategy in Triple-Negative Breast Cancer. Neoplasia.

[50] Nam A.-R., Jin M.-H., Bang J.-H., Oh K.-S., Seo H.-R., Oh D.-Y., Bang Y.-J. (2020). Inhibition of ATR Increases the Sensitivity to WEE1 Inhibitor in Biliary Tract Cancer. Cancer Res. Treat..

[51] Leibrandt R.C., Tu M.-J., Yu A.-M., Lara P.N., Parikh M. (2022). ATR Inhibition in Advanced Urothelial Carcinoma. Clin. Genitourin. Cancer.

